# Motor Training in Degenerative Spinocerebellar Disease: Ataxia-Specific Improvements by Intensive Physiotherapy and Exergames

**DOI:** 10.1155/2014/583507

**Published:** 2014-04-27

**Authors:** Matthis Synofzik, Winfried Ilg

**Affiliations:** ^1^Department of Neurodegenerative Diseases, Hertie-Institute for Clinical Brain Research, University of Tübingen, Hoppe-Seyler-Straße 3, 72076 Tübingen, Germany; ^2^German Research Center for Neurodegenerative Diseases (DZNE), 72076 Tübingen, Germany; ^3^Department of Cognitive Neurology, Hertie Institute for Clinical Brain Research, 72076 Tübingen, Germany; ^4^Centre for Integrative Neuroscience (CIN), 72076 Tübingen, Germany

## Abstract

The cerebellum is essentially involved in movement control and plays a critical role in motor learning. It has remained controversial whether patients with degenerative cerebellar disease benefit from high-intensity coordinative training. Moreover, it remains unclear by which training methods and mechanisms these patients might improve their motor performance. Here, we review evidence from different high-intensity training studies in patients with degenerative spinocerebellar disease. These studies demonstrate that high-intensity coordinative training might lead to a significant benefit in patients with degenerative ataxia. This training might be based either on physiotherapy or on whole-body controlled videogames (“exergames”). The benefit shown in these studies is equal to regaining one or more years of natural disease progression. In addition, first case studies indicate that even subjects with advanced neurodegeneration might benefit from such training programs. For both types of training, the observed clinical improvements are paralleled by recoveries in ataxia-specific dysfunctions (e.g., multijoint coordination and dynamic stability). Importantly, for both types of training, the retention of the effects seems to depend on the frequency and continuity of training. Based on these studies, we here present preliminary recommendations for clinical practice, and articulate open questions that might guide future studies on neurorehabilitation in degenerative spinocerebellar disease.

## 1. Introduction

The cerebellum is essentially involved in control of various kinds of motor behaviour such as speech, eye movements, limb movements, and balance. Here, its main function is the shaping and fine-tuning of movements. Correspondingly, cerebellar damage does not lead to reduced or paretic movements but to increased variability and poor accuracy of movements (“ataxia”) [1–3]. For example, ataxic gait is characterized by deficits like disordered coordination between head, trunk, and legs and impaired predictive postural adjustments, for example, impaired predictive postural adjustments [4] in balance control and multijoint leg coordination [5]. These deficits present as increased step width, variable foot placement, irregular foot trajectories, and a resulting instable stumbling walking path with very high movement variability [6–8] and a high risk of falling [9].

Given the fact that drug interventions are rare in degenerative diseases and limited to only specific type of diseases and symptoms [10], physiotherapy is a major cornerstone in current therapy of ataxic gait. However, while motor training programs have been shown to be beneficial in other neurodegenerative diseases (e.g., Parkinson's disease or stroke [11, 12]), their effectiveness remains controversial in the field of degenerative spinocerebellar ataxias [13–15]. Degenerative ataxias indeed seem to be the most difficult group of ataxias to treat. Here, motor training is not only challenged by the fact that the cerebellum is crucially involved in motor adaptation and motor learning [16–19] but is also challenged by the progressive nature of this type of disease and, in addition, by the fact that virtually all parts of the cerebellum are affected (although degeneration is frequently most prominent in the midline [20]). Moreover, degeneration in degenerative ataxias is mostly not limited to the cerebellum but often affects spinocerebellar pathways and dorsal columns as well [21]. In contrast, ataxia following stroke, neurosurgery, or trauma usually affects only circumscribed regions of the cerebellum but leaves other regions intact. These regions may compensate for the defective parts. In addition, in case of focal lesions, effects of neural plasticity are likely more effective because there is no competition with ongoing progressive neurodegeneration [22, 23]. Moreover, whereas patients with focal lesions clearly improve in motor functions over time [24], patients with degeneration slowly deteriorate [25]. Thus, in patients with progressive degenerative diseases, it would be a major achievement if they remained stable on the current status of motor function as long as possible or if progression of functional impairment was slowed down.

Until recently, only relatively few and small clinical studies have evaluated training interventions for patients with spinocerebellar ataxia. Using increasingly demanding balance and gait tasks, improvements were achieved in terms of increased postural stability and reduced dependency on walking aids [26, 27]. Locomotion training on treadmills with [28, 29] or without [30] body-weight support has been proposed, in particular for patients with more severe ataxia, which are not able to walk freely. However, many of these studies were single cases or based on a very small number of patients and did not focus on degenerative spinocerebellar ataxias but on nondegenerative secondary ataxias.

In clinical practice, this problem is complicated by the fact that not only the basis of scientific evidence for physiotherapy is sparse, but also the ataxia-specific expertise among physiotherapists. A large share of physiotherapists reports lack of ataxia-specific expertise and expresses the need for education and evidence-based guidelines [31, 32].

Here, we provide an analysis of the first recent clinical studies which have systematically investigated different training programs in sizeable cohorts of patients with degenerative cerebellar disease. Corroborated by encouraging findings from animal studies, which demonstrated motor learning effects in mice with degenerative cerebellar disease even on a molecular level [33], these studies provide the first systematic evidence for effectiveness of motor rehabilitation in this condition in humans [34–36]. Here, we review their findings, pinpoint their crucial advances and limitations, present recommendations for clinical practice, and articulate new research questions that might guide the field within the next years. This focused review might also facilitate first steps towards the development of evidence-based recommendations and specific education for physiotherapists.

## 2. Methods

*Search Strategy*. A selective, focused search was performed by the authors in the electronic databases of PubMed, Medline, and EMBASE. These databases were deemed most likely to cover all relevant clinical trials in this field and in particular to contain all studies that meet the stringent inclusion criteria mentioned below. Clinical trials were identified using a combination of the following terms and MeSH terms: cerebellar ataxia, ataxia, physiotherapy, physical therapy, training, exercise, and rehabilitation. The selected time period was from January 1, 1980, to December 18, 2013, and the articles had to be published in English. The retrieved articles were examined for useful references.

*Selection.* Articles were included if they met all of the following criteria: (i) original report, but not, for example, conference abstracts or reviews; (ii) prospective clinical trial evaluating the effectiveness of physical therapy or of other motor training programs focusing on gait and stance (e.g., computer assisted training, treadmill training, or videogame based training); (iii) high-intensity training over an extended time period, defined as repeated continuous exercises without interruption for at least >45 minutes per training session with ≥3 training sessions and ≥3 hours per week for ≥2 weeks; (iv) control design (case-control or intraindividual control design), but not uncontrolled case or cohort studies; (v) recruitment of patients with spinocerebellar degeneration, but not with secondary cerebellar ataxias due to, for example, stroke, tumor, trauma, inflammatory, or autoimmune causes (for a systematic review on training studies in these patients; see [37]). Spinocerebellar degeneration had to be a core feature in these patients. Studies were also included if degeneration of additional tracts was present, for example, dorsal columns or peripheral neurons, since this is the case in most degenerative spinocerebellar ataxias.

## 3. Results: Prospective Cohort Studies on Long-Term Motor Training in Degenerative Cerebellar Ataxia

We retrieved *n* = 578 studies using the combinations of terms and MESH terms mentioned above. *N* = 574 studies were excluded as they did not meet at least one of the five inclusion criteria. 3 studies were identified that investigated high-intensity motor training exercises in a sizeable cohort of patients with degenerative spinocerebellar ataxia (for an overview, see Table 1). These studies examined the following training strategies: physiotherapy combined with occupational therapy; physiotherapy focusing specifically on exercises challenging complex coordinative behaviors (“coordinative physiotherapy”); and training with whole-body controlled videogames (“exergames”). One additional study was identified that presented only a case report (on exergame-based training in advanced multisystemic degenerative ataxia), yet it did also meet the inclusion criteria.

### 3.1. Physiotherapy Training 

#### 3.1.1. Physiotherapy Combined with Occupational Therapy

Physiotherapy generally targets one or several of the following domains, often combining a mixture of them: balance, gait, coordination, strength, endurance, and posture [37]. One recent study combined a mixed multidomain physiotherapy strategy with occupational therapy, testing this intervention in 42 patients with cerebellar degeneration by a randomized controlled study design with delayed-entry of the control group [36]. Subjects were trained for an aggregated amount of 12 hours per week for 4 weeks. The authors observed improvements of ataxia severity, gait speed, fall frequency, and activities of daily living as determined by the Functional Independence Measure (FIM) [38]. More specific clinical ataxia assessment by the scale for the assessment and rating of ataxia (SARA) revealed an improvement of 2.1 points directly after the 4-week intervention (immediate and delayed-entry group aggregated). The SARA scale extends from 0 to 40 points, with higher scores indicating more severe ataxia [39]. The natural disease progression of degenerative cerebellar ataxias is 0.4–2.2 points per year on the SARA scale depending on genotypes [25]. This implies that the average improvement achieved by this kind of training is equivalent to gaining back functional performance of one or more years of disease progression. Improvements were more prominent in trunk ataxia than in limb ataxia, and patients with mild ataxia severity experienced a more sustained improvement in ataxic symptoms and gait speed [36]. Long-term follow-up data were collected up to 24 weeks after the intervention. Although functional status tended to decline to baseline level within this period, more than half of the patients retained an improvement in at least 1 item of the outcome measures at 24 weeks compared to baseline. Patients with sustained improvement had less severe ataxia (i.e., lower SARA score) than those without sustained improvement, indicating a possible predictive value of the SARA score—and thus ataxia severity—at baseline. Due to the study design, training intensity did not differ between subjects, thus making correlations between training intensity and training benefits impossible. No quantitative movement analysis was performed and the assessments in this study were not blinded.

#### 3.1.2. Coordinative Physiotherapy

One recent training study targeted specifically static and dynamic balance by a physiotherapy programme that focused on demanding coordinative exercises (“coordinative physiotherapy”) (for details on the exercises, see the following Table 4).

This strategy was tested in an intraindividual case-control design in 16 patients suffering from progressive ataxia due to cerebellar degeneration (*n* = 10) or degeneration of afferent pathways (*n* = 6) [34, 35]. Subjects were trained 1 hour per day, 3 days a week for 4 weeks under supervision of an ataxia expert physiotherapist at an ataxia centre, followed by 12-month home-training under the patient's own guidance.

The 4-week centre-based physiotherapy led to an improvement of 5.2 points on the SARA score directly after the intervention. This implies an average achievement equivalent to gaining back functional performance of at least two or more years of natural disease progression. Clinical assessments were additionally complemented by rater-independent quantitative movement analysis. This analysis revealed improvements in several aspects of gait like velocity or lateral sway as well as in the temporal and spatial variability of gait (e.g., step length, step cycle time). Variability in these measures has been discussed as a risk factor of falls in the elderly [40] as well as in subjects with cerebellar ataxia [41]. Moreover, it reduced temporal variability of intralimb coordination pattern in gait—a measure which has been shown to be specific for patients with cerebellar dysfunctions [8]. Patients with cerebellar ataxia profited more substantially from the intervention than patients with afferent ataxia. This discrepancy is most likely caused by a loss of afferent information in these patients, which removes necessary sensory inputs for adequate cerebellar processing.

**Figure 1:**
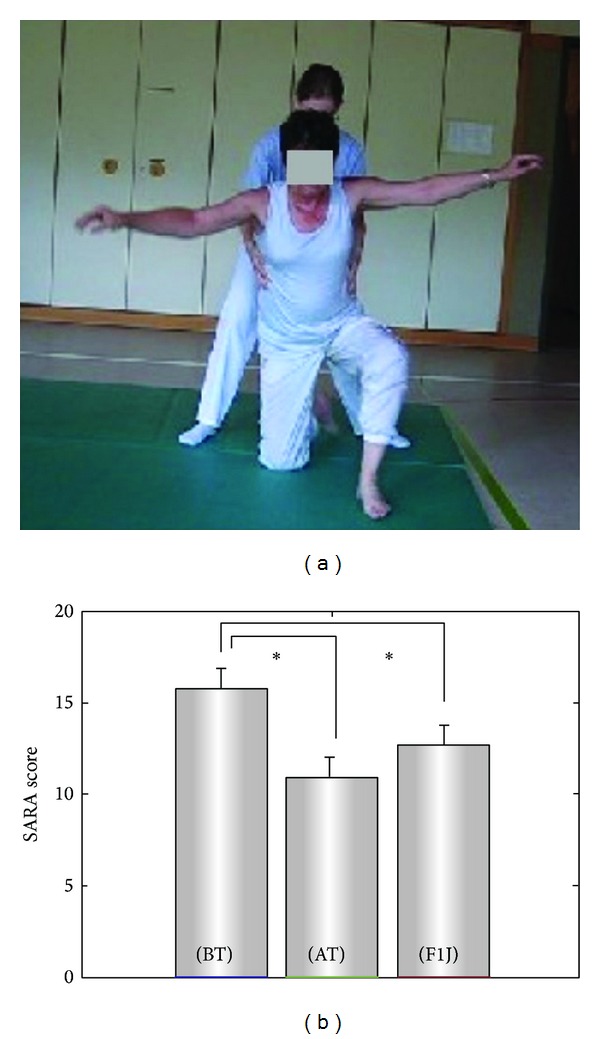
Coordinative physiotherapy. (a) Exemplary exercise of the training protocol: training of dynamic balance and multijoint coordination. (b) Group data of the clinical ataxia score SARA before training intervention (BT), after the four weeks training intervention (AT) and for follow-up assessment (F1J) after one year. Stars indicate significant differences between examinations (*P < 0.05). SARA: scale for the assessment and rating of ataxia ([35] reproduced with permission from Wiley).

Long-term effects and their translation to real-world functioning were assessed 12 months after the 4-week intervention period [35]. During these 12 months, subjects were trained by an individualized homework protocol combining different coordination exercises and degrees of difficulty, depending on the individual's level of functioning and learning success. Despite the underlying progression of the disease, SARA scores were still significantly better at this long-term followup than at baseline for the cerebellar group by 3.1 points (Figure 1). This indicates a retention of training effects that is equivalent to gaining back at least one or more years of natural disease progression. The group of afferent ataxia patients was stable compared to baseline. Independent from the type of ataxia, training intensity in coordination exercises correlated significantly with differences in SARA scores after 1 year, indicating that retention of training effects depends crucially on continuous training [35].

The goal attainment score (GAS) [42] was used to capture translation of training-induced effects into real-world functioning. For this score, each patient selects a personally meaningful goal reflecting an individually important activity of daily life (e.g., see Tables 2 and 3). These goals were determined before training and achievements were rated along the following Likert scale: “−2” = functioning like at baseline, “−1” = less than expected outcome, 0 = expected outcome, +1 = greater than expected outcome, and +2 much greater than expected outcome. For all patients, the average rating was 0.57, that is, above the expected level of achievement.

### 3.2. Exergame-Based Training

#### 3.2.1. Exergames Training in Mild-to-Moderate Degenerative Ataxia

Physiotherapy exercises might be complemented by (or used interchangeably with) whole-body training based on recently developed commercially available videogame technology (“exergames”). An exergame-based training strategy might have several advantages, in particular if used as a continuous long-term training for chronic diseases. (i) Exergame exercises involve highly motivational reward incentives and resort to diverse and stimulating exercise environments. (ii) Exergame-based training encompasses interactive exercises within rapidly changing environments, which could simulate and train patients' real-world activities and anticipatory coordination capacities. (iii) Patients with mobility impairments do not need to arrange access and transfer to external physiotherapy practices but can train within their own home environments.

**Figure 2:**
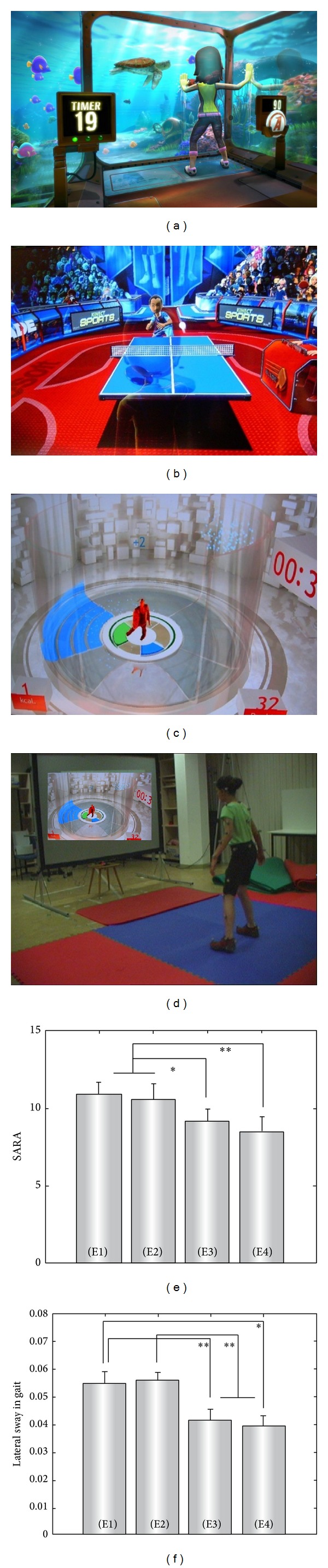
Exergame-based training. (a–c) Screenshots from the three XBOX Kinect games used in the training protocol. (a) 20000 leaks practice whole-body coordination and interaction with a dynamic environment; (b) table tennis practices goal-directed upper limb movements and dynamic balance, as well as movement timing; (c) light race practices goal-directed lower limb movements, fast movements, and dynamic balance. (d) Snapshot from the “Light Race” game. Patient C1 performs dynamic stepping movements in order to control the avatar to step onto the highlighted areas on the floor (figures reproduced with permission from Microsoft Xbox Kinect (a), (b) and Ubisoft (c), (d)). (e, f) Group comparisons of the clinical ataxia scores (SARA) and lateral sway in gait at examinations E1–E4. Patients were examined four times: two weeks before intervention (E1), immediately before the first training session (E2), after the two-week lab-training period (E3), and after the six-week home-training phase (E4) [43]. Stars denote significance: *P < 0.05, **P < 0.01.

Thus, taken together, exergames might present a novel, advantageous treatment tool for training patients with neurodegenerative diseases. It might allow patients to train coordinative exercises in a highly motivational and playful way at their own homes and with low financial costs. A directed exergames-based training program was recently investigated in 10 children with progressive spinocerebellar ataxia of a mild to moderate degree (i.e., all subjects were still able to walk without support) [43]. The investigators selected three commercially available Microsoft Xbox Kinect videogames, which were specifically chosen to target motor capacities known to be dysfunctional in ataxia, namely, goal-directed limb movements, dynamic balance, and whole-body coordination (Figure 2). The training program started with a 2-week laboratory training phase individually supervised and directed by a physiotherapist, who introduced the games and adequate movement strategies to the subjects. During these two weeks, subjects were trained 1 hour per day, 4 days a week. This initial training phase was followed by 6 weeks of home-training phase during which the patients were asked to continue the exergame-based exercises at home. Effects of the training were assessed in an intraindividual control design. SARA ratings were performed in a blinded fashion, which was achieved by presenting videos of single SARA examinations of individual subjects in a random fashion to a rater who was blinded to the number of the specific examination. These rater-blinded assessments revealed a reduction by 2 SARA points on average after 8 weeks of training (Figure 2), indicating an achievement that is equivalent to gaining back at least one or more years of natural disease progression. The improvements in ataxia during the home-training were thereby dependent on the intensity of home-training: the more intensive the training periods at home, the higher the reduction in SARA gait and posture. The clinical improvement in ataxia severity was paralleled by improvements in quantitative measures of gait (lateral sway, step length variability) [43] (Figure 2) and, even more importantly, by improvements in ataxia-specific dysfunctions like multijoint coordination and dynamic stability [44]. This included complex whole-body movements highly relevant for everyday living, for example, rapid stepping movements to compensate for gait perturbations and to prevent falls. Improvements transferred also to other movements, indicating a generalization effect of the underlying control mechanisms induced by exergames training [44]. These findings demonstrate that exergames training yields a specific effect on ataxia and dynamic balance which goes beyond a mere improvement in subjects' game scores, motivation, and fitness. Training was highly motivational for all participating subjects throughout the whole training period [43].

In sum, this study suggests that directed training of whole-body controlled videogames might present a highly motivational, cost-efficient, and home-based rehabilitation strategy to train dynamic balance and interaction with dynamic environments for subjects with chronic coordination disturbances.

#### 3.2.2. Exergames Training in Advanced and Multisystemic Ataxia

Exergame-based training might improve coordination in subjects with mild-to-moderate spinocerebellar ataxia. Yet, it is still an open question whether it is also effective in subjects with advanced degenerative cerebellar disease who are already wheelchair-bounded and, moreover, where ataxia is part of a multisystemic disease affecting many additional pathways of the central and peripheral nervous system. These subjects are largely considered to benefit only poorly from treatments which is indicated, for example, by the fact that they are commonly excluded from current drug treatment trials [45, 46], thus leaving them without prospects of access to novel treatments.

A recent case study provided first proof-of-principle evidence that exergame-based coordinative training might indeed serve as an effective treatment even for advanced, multisystemic degenerative ataxia [47]. The investigators used a sequentially structured 12-week coordinative training program based on specifically selected, commercially available Nintendo Wii games in a child with advanced ataxia telangiectasia (AT) who was already largely wheelchair-bounded. Outcomes were assessed in a rater-blinded intraindividual control design. The authors observed an improvement of 4.4 points on the SARA scale, which was most pronounced in posture and residual gait function. Correspondingly, subjective achievement ratings in the GAS show marked balance improvements in sitting and stance [47]. These results seem to indicate that, despite advanced multisystemic disease (including oculomotor and cognitive deficits), exergame-based coordinative training might lead to substantial effects which translate into daily living. However, these preliminary results need to be confirmed in a larger cohort study before firm conclusions can be drawn.

## 4. Discussion

The above described studies provide first evidence for sizeable cohorts that high-intensity motor training might be effective in degenerative ataxia. More specifically, they provide proof-of-concept evidence that

1. patients with degenerative ataxia benefit from coordinative training which might be based either on physiotherapy or on exergames;
2. improvements are equal to regaining 1 or more years of natural disease progression;
3. improvements are not due to unspecific changes but due to recoveries in ataxia-specific dysfunctions;
4. retention of training effects depends on the continuity of training;
5. even subjects with advanced neurodegeneration benefit from these therapies;
6. even children with severe disease can be highly motivated to train throughout the whole demanding program and that they experience feelings of success about their own movements.

### 4.1. Open Questions and Future Studies

Albeit promising, the aforementioned studies have important limitations. These limitations stimulate new research questions that might guide the field in the next years. Larger cohort studies are warranted to confirm the aforementioned findings. As degenerative ataxias belong to “orphan diseases” with a prevalence of approximately 6 : 100.000 [48], it will require coordinated multicenter efforts to aggregate larger cohorts. These cohorts should be more homogeneous. The phenotypic and genetic variability between different degenerative ataxias is large, including different disease progressions and different comorbid affection of additional neural systems [21, 49, 50]. Thus, future studies should ideally resort to cohorts with prespecified, homogenous genotypes. Moreover, they should aim at using a randomized control design to yield higher levels of evidence. The intraindividual control design used by three of the four aforementioned studies [34, 43, 47] has several attractive advantages, since subjects are here taken as their own controls and thus between-group differences in disease progression and comorbid affection of different neural systems can be ruled out. However, a randomized control design is still methodologically superior, and a delayed-entry design as employed in the study of Miyai and colleagues [36] guarantees that also the control group will receive the benefits of motor training. Moreover, studies should use a multicenter design, as only this design could prove that the specific training is indeed transferable to other centers and therapists.

Future studies should also focus more specifically on identifying predictors for training success. The type of ataxia might serve as a predictor, as, for example, indicated by the finding of Ilg and colleagues that patients with afferent ataxia benefit less than patients with cerebellar ataxia [34, 35]. However, this might not generally be true as younger patients with afferent ataxia (namely, Friedreich's ataxia) still benefitted well from XBOX-based exergame training [43]. Another predictor might be severity of ataxia at baseline, as suggested by the finding of Miyai and colleagues that patients with more severe ataxia had less sustained improvement by training [36]. But, again, this might not generally be true, as indicated by the finding of Synofzik and colleagues that a wheelchair-bound subject with advanced degenerative ataxia still achieved a remarkable improvement of 4.4 points on the SARA scale [47]. Finally, the specific level of residual cerebellar integrity might be a predictor of the capacity to improve motor performance. Studies from subjects with focal cerebellar lesions (e.g., due to stroke or tumor) have indicated that in particular the integrity of the deep cerebellar nuclei might determine future rehabilitation success [51, 52].

The changes in neural mechanisms and substrates underlying the training effect in degenerative ataxias are still largely unclear. Is the degenerating cerebellum still able to adapt motor coordination or, instead, is the learning deficit compensated by other brain structures? In an attempt to depict the brain changes that contribute to improvement of motor function, Burciu et al. [53] performed a voxel-based morphometry (VBM) study in patients with cerebellar degeneration. A two-week postural training resulted in a significant improvement of balance in degenerative patients. Comparing gray matter volumes before and after training revealed an increase primarily within nonaffected neocortical regions of the cerebellar-cortical loop, more specifically the premotor cortex. Gray matter changes were observed within the cerebellum as well but were less pronounced. Thus, these first data suggest that training may lead to activation and plasticity of compensatory networks and, to a smaller extent, even of remaining cerebellar circuitry itself. Further imaging studies on neurorehabilitation strategies will lead to a better understanding of the underlying pathomechanisms of disordered motor performance and learning. This might help to tailor physiotherapy to the specific needs of patients with cerebellar ataxia.

### 4.2. Preliminary Implications for Practice: Physiotherapy versus Exergames—A Wrong Dichotomy

Despite their limitations, the aforementioned studies provide some preliminary hints on the relation between physiotherapy versus exergame training in long-term training protocols. These hints stimulate future investigations, validating these ideas and exploring them in more detail.

The experience from these studies indicates that exergame-based training can and should not replace physiotherapy. It might rather serve to complement physiotherapy-based programs by helping subjects to achieve and maintain the required training intensity even over a long training period and by practicing specific coordination skills such as rapid adaptation to dynamically changing environments and updating of predictions to novel external events. These skills are highly needed in real-world situations where subjects have to adequately react under time pressure to constantly changing environmental conditions and to accurately anticipate novel events and their impact for one's own sensorimotor system. It was shown that both of these skills are impaired in degenerative cerebellar disease [54, 55], yet they are less trained by conventional physiotherapy compared to exergames. Due to the training in an interactive, constantly changing environment with many novel unexpected events, exergames better simulate real-world situations. Well-selected games (Figure 2) allow to efficiently improve these highly valuable coordination skills, as shown recently [43, 47, 56].

However, the experience from the abovementioned studies also reveals that any exergame-based training needs to be initiated and supervised to a variable degree by a physiotherapist. The specific expertise of this professional is needed to select appropriate exergames according to each individual's coordinative capacities, degree of impairment, and current treatment goals. Only this selection and supervision can ensure that the patient is neither over- or underchallenged which would quickly lead to a lack of motivation. Moreover, it ensures that the patient is not predisposed to risky movements which might lead to falls and other harms. Finally, it seems preferable that, at least during the first training sessions, the physiotherapist would actively coach the patient how to make adequate complex coordination movements when trying to solve the exergame challenges. We have observed that ataxia patients initially often try to play these games by reducing their movement repertoire even more, trying to keep their center of mass within their base of support by making themselves “stiff” and by actually avoiding complex movements [43, 44]. If entrenched by continued playing, this derivative compensatory movement strategy might lead to a further* loss *of coordination skills, that is, trigger a downward spiral, thus indicating that exergame-based training might even be* harmful *if not applied adequately. A parallel training and supervision by a physiotherapist likely help the patient to avoid such wrong movement strategies and to relearn to make complex movement sequences, even if they seem risky in the beginning.

### 4.3. Recommendations for Clinical Practice

Based on the aforementioned studies, a new concept for ataxia training in clinical practice emerges. This concept still has to be validated in practice and should thus be seen as a preliminary rather than a definitive suggestion. Yet it might stimulate future research and clinical rehabilitation practice.

This concept is characterized by the idea that rehabilitation in degenerative ataxias should optimally resort to a large array of different training strategies which should be individually tailored according to each individual's ataxia type, disease stage, and personal training preferences. In very* early stages of ataxia*, even demanding sportive exercises might be selected which place high challenges to the coordination system, for example, table tennis, squash, or badminton. These real-world sports might be complemented by demanding XBOX Kinect games (e.g., “Light Race” or “20.000 Leaks”) or Wii games (e.g., “PhysioFun”). These games might be played on an elastic mattress to increase the coordinative challenge even more. In* mild-to-moderate ataxia stages*, a coordinative physiotherapy program under the guidance and supervision of a professional physiotherapist receives major importance [34]. This might include the training of secure fall strategies in addition of training to avoid falls. The exergame-based training component might switch to a little less challenging XBOX or Wii games, for example, “tightrope walk” or “ski slalom.” In* advanced ataxia stages*, no clear evidence-based physiotherapy training program does yet exist. However, in severe cases, in which free standing and walking is not possible anymore, treadmill training [28–30] with potential weight support may be helpful to increase walking capabilities (with the use of mobility aids) and to preserve general fitness as far as possible [10]. The exergame-training component has to be limited to Wii games, as XBOX Kinect games cannot be played by subjects who are bound to a sitting position. Here, subjects will be seated onto the Wii balance platform which then serves to detect shifts of weight of the trunk. Candidates for appropriate Wii games include “penguin slide,” “table tilt,” or “bubble balance” [47]. Taken together, such individualized tailored training strategies might help to maximize the function of each individual subject in his or her particular disease state, and might—at least in some cases—slow down a possible downward-spiral of ataxia-related immobility and further deterioration of coordinative functions.

In conclusion, rehabilitation for degenerative cerebellar disease will remain a challenge for patients, physicians, and therapists. However, recent advantages in both clinical rehabilitation and research on motor adaptation in cerebellar disease will stimulate further studies and hopefully lead to broader knowledge in this challenging field of motor rehabilitation and finally to an improvement of the patients' quality of life.

## Figures and Tables

**Table 1:** Overview of high-intensity training studies in degenerative ataxia.

	Physiotherapy combined with occupational therapy [36]	Coordinative Physiotherapy [34, 35]	Exergames training [43]
Number of patients	42	16	10

Type of disease	SCA6 (20), ADCA (6), and IDCA (16)	SCA6 (2), SCA2 (1), ADCA (1), IDCA (6), FRDA (3), SANDO (2),and SN (1)	FRDA (4), arCA (3), AOA2 (1), and ADCA (2)

Age ± SD (range)	62.5 ± 8.0 (range: 40–82)	61.4 ± 11.2 (range: 44–79)	15.4 ± 3.5 (range: 11–20)

Gender	22 males, 20 females	8 males, 8 females	5 males, 5 females

Duration of disease	9.8 ± 6.2 (7 months–30 years)	12.9 ± 7.8 (3–25 years)	

Baseline SARA	11.3 ± 3.8 (5–21.5)	15.8 ± 4.3 (11–24)	10.9 ± 2.3 (7–13.5)

Control	Crossover for short-term effect	Intraindividual controls for short-term effect	Intraindividual controls

Evidence class	Class Ib	Class III evidence	Class III evidence

Intervention	2 hours × 5 days + 1 hour × 2 days per week for 4 weeks	1 hour, 3 days per week for 4 weeks	1 hr × 4 per week for 2 weeks at lab; variable frequency at subjects' own motivation for 6 weeks at home

After training	No	Home-training protocols	No

Outcome measures	SARA, FIM, gait speed, cadence, FAC, and falls	SARA, gait speed, balance, BBS, GAS, and movement analysis	SARA, balance, ABC scale, DGI scale, GAS, and movement analysis

Assessment point	Baseline, post 0, 4, 12, and 24 weeks	4 weeks pre, baseline, and post 0, 8 weeks	2 weeks pre, baseline, and post 0

Main results	SARA and gait improved 12 wks but not 24 wks	SARA and gait improved 8 wks after rehabilitation only in patients with cerebellar ataxia not afferent ataxia	SARA and gait improved directly post rehabilitation; improvement correlated with individual's training intensity at home

SCA: spinocerebellar ataxia; FRDA: Friedreich's ataxia; IDCA: idiopathic cerebellar ataxia; ADCA: autosomal dominant cerebellar ataxia of unknown type; SANDO: sensory ataxic neuropathy with dysarthria and ophthalmoparesis caused by mutations in the polymerase gamma gene; SN: sensory neuropathy with cerebellar degeneration; arCA: autosomal recessive cerebellar ataxia of unknown type; AOA2: ataxia with oculomotor apraxia type 2; SARA: scale for the assessment and rating of ataxia; ABC: activity-specific balance confidence scale; BBS: Berg balance score; GAS: goal attainment scaling [42]; DGI: dynamic Gait index; FIM: functional independence measure [38]; and FAC: functional ambulation categories. Evidence was graded according to the Oxford Center for Evidence Based Medicine (CEBM) classification. This table presents details of the first three clinical studies of motor rehabilitation in larger cohorts in degenerative spinocerebellar disease [34–36, 43].

**Table 2:** Personally selected goal of the goal attainment score for an exemplary individual with degenerative cerebellar ataxia (subject C4).

Individual goal: walking around a table with small distance without swaying	Score
The patient walks around the table mainly by touching the table	−2
The patient can walk around the table without touching the table most of the time	−1
The patient can walk around the table without touching the table	0
The patient can walk around the table without touching the table and he is able to look around sometimes	+1
The patient can walk around the table without touching the table and he is able to look around the whole time	+2

Five levels of goal attainment were defined before the intervention started. Scores range from −2 to 2 (−2~baseline, −1~less than expected outcome, 0~expected outcome, 1~greater than expected outcome, and 2~much greater than expected outcome [35]).

**Table 3:** Personally selected goals of the goal attainment scale and the scores obtained after the intervention period.

Patient	Goal	Score
C1	Walking on a narrow path (<50 cm)	2
C2	Walking up a staircase without using railway	2
C3	Reaching the mailbox in a distance of 600 without using a walking aid	0
C4	Walking around a table with small distance without swaying	1
C5	Walking without a walking aid over a distance >10 m	1
C6	Walking over a distance of about 300 m without a walking aid or a helping person	2
C7	Walking over a distance of 50 m with a trolley, without bumping with the feet into it	1
C8	Walking free on a small staircase (3 steps) in an alternating way with a distance of 1 m to the railway	−1
C9	Walking with a trolley over a distance of 50 m	0
C10	Walking without a walking aid over a distance of about 100 m	0
A1	Walking independently over longer distances (>500 m)	1
A2	Reducing danger of falling	0
A3	Walking a distance of 30 m with a full cup without spilling something	−1
A4	Walking with a trolley over a distance of 2000 m without dropping feet and strong support from the arms.	−1
A5	Walking over a distance of 100 m with a trolley and without bumping with the feet into it	2
A6	Walking with a trolley over a distance of 500 m	−1

Described goals correspond to score 0. Scores range from −2 to 2 (−2~baseline, −1~less than expected outcome, 0~expected outcome, 1~greater than expected outcome, and 2~much greater than expected outcome [35]).

**Table 4:** Exercises of the coordinative physiotherapy program.

Static Balance
(i) Standing on one leg.
(ii) Quadruped standing: stabilize the trunk and lift one arm.
(iii) Quadruped standing: stabilize the trunk and lift one leg.
(iv) Quadruped standing: lift one arm and the leg of the other side.
Dynamic Balance
(i) Kneeling: put one foot in front and back alternately.
(ii) Kneeling: put one foot to the side and back alternately.
(iii) Kneeling: put one foot in front, stand up, and put one leg back with kneeling alternately.
(iv) Standing: swing arms, see saw knees.
(v) Standing: step to the side.
(vi) Standing: step in front.
(vii) Standing: step back.
(viii) Standing: cross over step.
(ix) Climbing stairs.
(x) Walking over uneven ground.
Whole Body Movements to Train the Trunk-Limb Coordination
(i) Quadruped standing: lift one arm and the leg of the other side, flex arm, leg, and trunk, and extend arm, leg, and trunk alternately.
(ii) “Morning prayer” (Moshe Feldenkrais): kneeling: bend legs, arms, and trunk (“package sitting”): extend legs, arms, and trunk alternately.
(iii) Kneeling: sit beside the heel on the right side; kneeling: sit beside the hell on the left side alternately.
Steps to Prevent Falling and Falling Strategies In Order To Prevent Trauma
(i) Standing: step to the side, step in front, step back, and cross over step in a dynamic alteration.
(ii) Standing: the therapist pushes the patient in altered directions; the patient has to react quickly with fall preventing steps.
(iii) Standing: bend the trunk and the knees to touch the floor and erect the body alternately.
(iv) Standing: bend the trunk and the knees, touch the floor, and go down to quadruped standing,
(v) Standing: the therapist pushes the patient; the patient has to react quickly-bend and go to the floor in a controlled manner
(vi) Walking—the therapist pushes the patient—the patient has to react quickly, bend, and go to the floor in a controlled manner.
Movements to Treat or Prevent Contracture Especially Movements of Shoulders and Spine
(i) Extension of the spine: prone lying: push up the shoulder girdle from prone lying; prone lying on a wedge.
(ii) Rotation of the spine: supine lying: knees are bended, rotate the knees to the right and left side,
(iii) Flexion of the shoulder: supine lying: lift the arms in the direction of the head.

## Abbreviations

GAS: goal attainment score
SARA: scale for the assessment and rating of ataxia.
